# Interpretable machine learning for cognitive impairment assessment: integration of clinical and radiomic white matter hyperintensities features

**DOI:** 10.1186/s12967-026-07843-6

**Published:** 2026-02-11

**Authors:** Mengchen Wang, Tianci Wang, Xiaoxiao Wang, Chun Liu, Frankliu Gao, Bensheng Qiu, Tao Guo, Yu Huang

**Affiliations:** 1 Department of Medical Imaging Technology, School of Medical Imaging, Bengbu Medical University, Bengbu, Anhui 233030 China; 2 Medical Imaging Center, Department of Electronic Engineering and Information Science, University of Science and Technology of China, Hefei, Anhui 230026 China; 3 The First Affiliated Hospital of the University of Science and Technology of China, Hefei, Anhui China; 4 Michael G. Foster School of Business, University of Washington, Seattle, WA Box 353200 98195-3200 USA

**Keywords:** White matter hyperintensities, Radiomics, Clinical data, Machine learning, Cognitive impairment

## Abstract

**Background:**

White matter hyperintensities (WMH) are recognized as important imaging biomarkers associated with cognitive impairment. This study aimed to develop a machine learning model that combines clinical data and WMH radiomic features for improving the assessment of cognitive impairment

**Methods:**

A retrospective study was conducted on 303 patients with WMH. Clinical data and magnetic resonance imaging scans were collected, and cognitive function was evaluated using the Montreal Cognitive Assessment (MoCA). WMH lesions were segmented on T2 Fluid-Attenuated Inversion Recovery images using the SAM2UNET model, followed by the extraction of radiomic features from the segmented WMH regions. After feature selection with LASSO and recursive feature elimination (RFE), six machine learning models were developed, and the optimal model was identified. SHapley Additive exPlanations (SHAP) were applied to enhance the interpretability of the model. Model performance was evaluated using metrics such as the area under the receiver operating characteristic curve (AUROC), accuracy, precision, recall, and F1-score.

**Results:**

The integrated TabPFN model, utilizing 10 clinical and 5 radiomic features, achieved the superior overall predictive performance. The model yielded an AUROC of 0.842, an F1-score of 0.737, an accuracy of 0.754, a recall of 0.750, a precision of 0.724, and a specificity of 0.758, respectively. Calibration and decision curve analyses indicated good agreement and favorable clinical utility of the model in assessing cognitive impairment.

**Conclusion:**

This study established a reliable and interpretable TabPFN model integrating routine clinical and radiological data, offering a promising tool for the early detection and personalized management of cognitive impairment in community populations.

## Introduction

Cognitive impairment (CI) represents a spectrum of neurocognitive decline that poses a major public health challenge in aging societies, with prevalence markedly increasing in individuals aged over 60 years [1, 2]. It is widely regarded as an intermediate stage between normal aging and dementia, particularly Alzheimer’s disease (AD) [3]. Epidemiological evidence indicates that approximately 10–15% of patients with mild CI progress to AD annually, while only a small proportion of the general elderly population develops dementia during the same period [4–6]. As CI develops, patients typically experience irreversible impairments in memory, language, calculation, and executive functions, often accompanied by mood disturbances and behavioral abnormalities [7]. In advanced stages, patients may lose the ability to perform activities of daily living, leading to increased caregiver burden and mortality risk [8]. Therefore, early identification and accurate assessment of CI are essential for slowing disease progression and informing effective intervention strategies.

Currently, the clinical assessment of CI primarily relies on a comprehensive evaluation that integrates medical history, neuropsychological tests, neuroimaging, and fluid biomarkers [9]. Firstly, detailed medical history taking can facilitate understanding of the timing, characteristics, and possible risk factors of cognitive decline, providing basic information for preliminary diagnosis. Next, neuropsychological tests such as the Mini-Mental State Examination (MMSE) and the Montreal Cognitive Assessment (MoCA) are the most commonly used screening tools, exhibiting moderate diagnostic sensitivity and specificity [10, 11]. Thirdly, among neuroimaging modalities, magnetic resonance imaging (MRI) and computed tomography (CT) play a central role in detecting structural and functional abnormalities such as cortical atrophy, white matter lesions, and cerebrovascular injury [12]. In contrast, fluid biomarkers (e.g., β-amyloid and tau proteins in cerebrospinal fluid) provide molecular-level evidence of neurodegeneration but are costly, invasive, and technically demanding, limiting their feasibility for large-scale screening [13].

MRI has offered a noninvasive and reproducible means to visualize brain structural and microvascular alterations. It has become indispensable in CI research owing to its high spatial resolution and multimodal capability. Among MRI findings, white matter hyperintensities (WMH) are among the most common age-related changes and are widely recognized as imaging hallmarks of cerebral small vessel disease (CSVD) [14]. Increasing evidence demonstrates that WMH burden is strongly correlated with cognitive deficits, particularly in attention, executive, and processing-speed domains [15]. Research indicates that the spatial distribution of lesions, such as periventricular and deep WMH, and pathological heterogeneity, including chronic hypoperfusion, demyelination, and neuroinflammation, play critical roles in cognitive function [16–18]. Therefore, WMH imaging features may aid in the early identification of individuals at high risk for CI. While MRI is highly effective in detecting CI-related brain structural changes, traditional assessments rely on radiologists’ subjective evaluations, thereby limiting the ability to objectively and reliably quantify subtle and heterogeneous tissue alterations.

In recent years, driven by the rapid development of artificial intelligence and advanced imaging analysis techniques, radiomics has emerged as a high-throughput computational approach capable of extracting numerous quantitative imaging features beyond human visual perception [19]. The combination of radiomics and machine learning (ML) facilitates the identification of imaging biomarkers associated with cognitive impairment, contributing to a more objective and quantitative evaluation process. Previous studies applying ML algorithms such as support vector machines (SVM), random forests (RF), and logistic regression (LR) have demonstrated promising performance in distinguishing CI or predicting its progression [20–22]. Although WMH is recognized as a high-risk factor for CI, most existing studies have focused on patients with AD, and systematic investigation of early CI detection in the general elderly population with WMH remains limited.

**Fig. 1:**
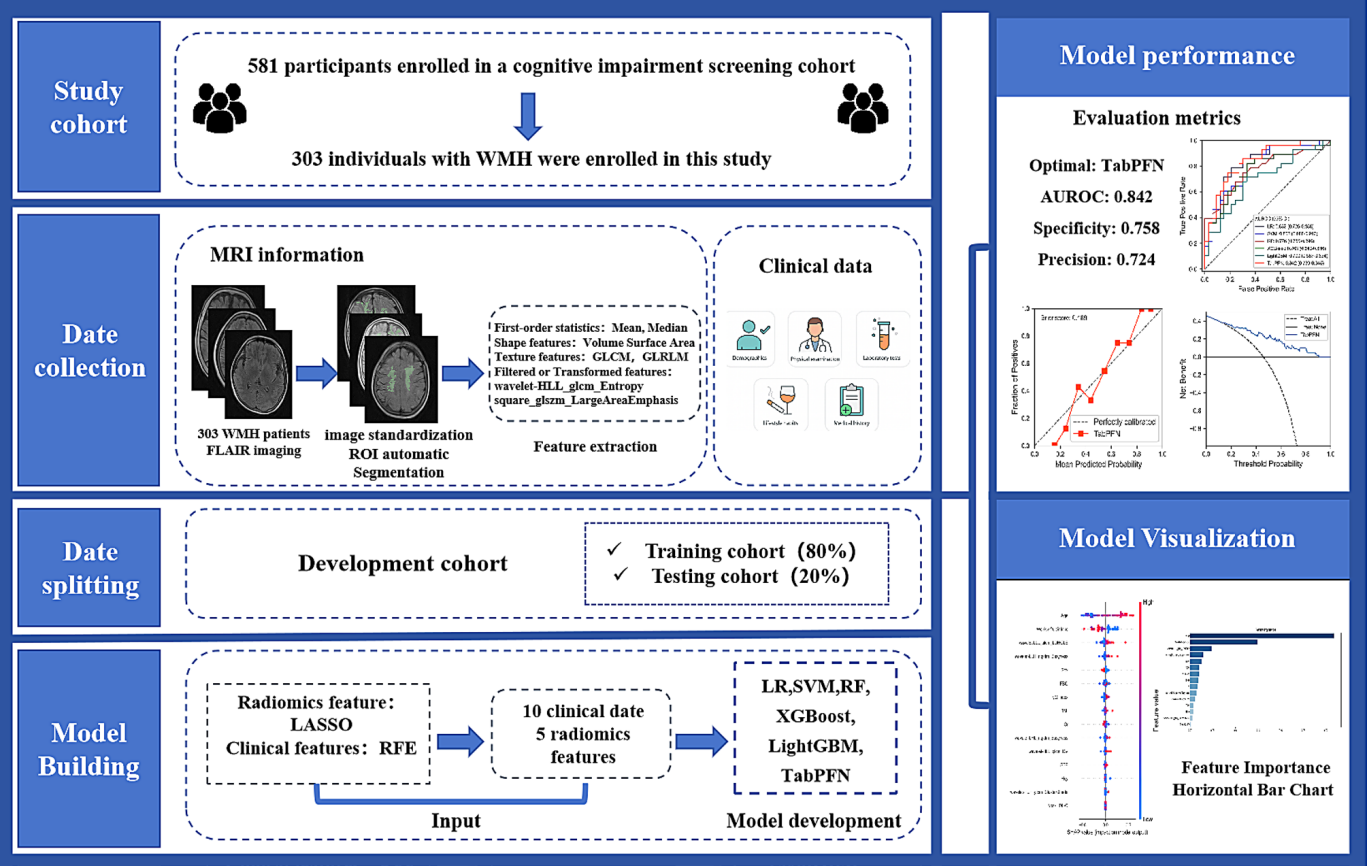
Overview of the study design. The figure illustrates the study cohort selection, MRI data collection and feature extraction, dataset splitting into training and testing cohorts, machine learning model development using radiomic and clinical features, and model evaluation, including performance metrics and feature importance visualization. The left side illustrates the development process of the machine learning model for cognitive impairment assessment, while the right side shows the model evaluation and visualization. Abbreviations: ROI, region of interest

To address these limitations, this study integrates WMH radiomic features with clinical data and employs ML algorithms to construct an assessment model for CI, as illustrated in Fig. 1. The goal is to enhance the accuracy of CI evaluation, enable efficient identification, and improve early screening and diagnostic capabilities.

## Materials and methods

### Patients

This retrospective cross-sectional study analyzed data from 581 participants enrolled in a cognitive impairment screening cohort conducted in Guizhou, China, between October 23 and November 8, 2023, at the First Affiliated Hospital of the University of Science and Technology of China. Participants were enrolled according to the following inclusion and exclusion criteria: Inclusion criteria were defined as follows:(1) Age ≥ 40 years;(2) Ability to complete the MoCA and cooperate with relevant examinations; (3) Imaging findings consistent with the characteristics of WMH as defined by the STRIVE-2 criteria [14]. Exclusion criteria included: (1) Acute intracranial large vessel lesions, such as ischemic stroke or intracerebral hemorrhage; (2) Non-vascular white matter lesions, including immune-mediated demyelination, hypoxic-ischemic encephalopathy, metabolic or toxic encephalopathy; (3) Coexisting intracranial pathologies, such as traumatic brain injury or tumors; (4) Incomplete clinical or imaging data; (5) Poor image quality without diagnostic value; (6) Absence of WMH findings on MRI. The Institutional Ethics Committee of the First Affiliated Hospital of the University of Science and Technology of China approved this study. Given its retrospective nature, the requirement for informed consent was waived.

### Data collection

In the baseline survey, all participants completed the MoCA questionnaire and were subsequently classified into the cognitively normal group and the cognitive impairment group. Additionally, all participants underwent physical and laboratory examinations. Physical examinations included weight, abdominal circumference (AC), systolic blood pressure (SBP), and diastolic blood pressure (DBP). Laboratory tests collected indicators such as fasting blood glucose (FBG), triglycerides (TG), total cholesterol (TC), low-density lipoprotein cholesterol (LDL-C), high-density lipoprotein cholesterol (HDL-C), homocysteine (Hcy), uric acid (UA), and creatinine (Cr). Calculations for triglyceride glucose index (TyG index), body mass index (BMI), and non-high-density lipoprotein Cholesterol (Non-HDL-C) were also performed. Demographic characteristics and lifestyle habits were additionally recorded.

### Assessment of cognitive impairment

Cognitive impairment refers to difficulties in domains encompassing memory, language, attention, reasoning, planning, or problem-solving that impair daily functioning and independence [23]. The MoCA was used for evaluation, as it is a well-validated, sensitive tool for screening subtle cognitive deficits [24, 25]. The MoCA covers multiple cognitive domains, including visuospatial/executive function, naming, attention, language, abstraction, delayed recall, and orientation, with a maximum score of 30. Given that the study population consisted primarily of older adults, and based on prior research in Chinese elderly populations, a cutoff score of 24 was applied to categorize participants into cognitive impairment ( < 24) and no cognitive impairment (≥24). Several high-quality studies support a cutoff at or around 24. For example, a systematic review and meta-analysis reported that MoCA cutoffs of < 24 maximize the sum of sensitivity and specificity for detecting cognitive impairment [26]. A 2023 meta-analysis in Chinese elderly populations found that 24/25 provides diagnostic value nearly equivalent to 25/26 [27]. In a clinical sample, a revised cutoff of 23/24 offered higher specificity than the original 25/26 recommendation [28].

### MRI image acquisition

This study utilized a mobile 0.35T MRI scanning system (CLIMBER035) from Anhui Fuqing Medical Technology Co., Ltd., equipped with a standard 4-channel head coil for image acquisition. The scanning position was designed as follows: patients were placed in a supine position with both hands resting at their sides, and the positioning center was aligned with the coil center and the glabella. During scanning, an axial T2 Fluid-Attenuated Inversion Recovery (FLAIR) sequence was acquired and the scanning parameters were as follows: TR = 7200 ms, TE = 90 ms, slice thickness = 6 mm, echo train length = 8, FOV = 240 × 240 mm, slice gap = 7.5 mm.

### WMH segmentation and feature extraction

Since the FLAIR sequence is particularly sensitive to WMH detection, segmentation, and feature extraction of WMH were performed on FLAIR images to achieve precise delineation of WMH regions. An advanced deep learning model, SAM2UNET, was employed, which combines the segmentation capability of the Segment Anything Model (SAM) with the fully automated segmentation framework of U-Net, achieving highly efficient and completely automated segmentation that significantly reduces manual intervention [29]. The SAM2UNET model is based on the classical U-shaped network architecture, enhanced by incorporating a Receptive Field Block (RFB) module at each skip connection to capture multi-scale image features. The decoder consists of a trainable, simple two-layer convolutional network. Utilizing the U-Net architecture, the model generates segmentation maps after multiple decoding stages, providing accurate delineation of WMH regions in FLAIR images [30].

To evaluate the performance of the SAM2UNET model for WMH segmentation, a dataset comprising both proprietary and publicly available samples was assembled for training and validation. The dataset comprised 135 subjects, including 77 internally annotated samples from the company’s proprietary dataset (CLIMBER035) and 58 publicly available samples acquired from 3.0T MRI scans. The data were split into training and testing cohorts at a ratio of 10:1. Following 300 epochs of training, the model achieved a Dice coefficient of 0.85 on the testing cohort, demonstrating high accuracy and robustness in WMH segmentation. Radiomics feature extraction was conducted using the Pyradiomics package in Python, automatically extracting radiomic features from the WMH regions delineated on the FLAIR images.

### Feature selection

For the radiomics features, the least absolute shrinkage and selection operator (LASSO) regression was applied to identify the most informative variables associated with CI. This method introduces an L1 regularization penalty to shrink less important feature coefficients toward zero, thereby achieving both feature selection and dimensionality reduction. For the clinical variables, recursive feature elimination (RFE) was applied to iteratively rank feature importance and remove less informative variables until optimal model performance was achieved. This approach ensured that only the most robust and clinically relevant features were retained in the final model.

### Machine learning algorithms

The machine learning algorithms used in this study include LR, SVM, RF, Extreme Gradient Boosting (XGBoost), Tabular Prior-Data Fitted Networks (TabPFN), and Light Gradient Boosting Machine (LightGBM).

LR is a classical statistical model widely used for binary classification, which estimates the probability of a given outcome based on a linear combination of input features and applies the logistic function to model the log-odds of the target event.

SVM was employed to classify cognitive impairment status by constructing hyperplanes in the feature space that maximize the separation margin. Kernel transformations allowed the model to capture complex, non-linear relationships among imaging and clinical features.

RF is an ensemble method that aggregates multiple decision trees trained on bootstrapped subsets of the data, employing random feature selection at each split to reduce overfitting and enhance generalizability.

XGBoost is an optimized gradient boosting framework that iteratively fits decision trees to correct residual errors, incorporating regularization and parallelization to improve predictive accuracy and computational efficiency on large datasets.

LightGBM advances this approach by using a leaf-wise growth strategy and innovations such as Gradient-Based One-Side Sampling and Exclusive Feature Bundling, enabling faster training and improved handling of high-dimensional data.

TabPFN leverages Bayesian deep learning principles to approximate posterior predictions from tabular data, requiring virtually no hyperparameter tuning and providing a flexible and theoretically well-founded approach for probabilistic classification.

### Development and evaluation of machine learning models

The dataset was randomly split into a training cohort (80%) and a testing cohort (20%). Six ML algorithms were employed to develop models incorporating both clinical and radiomic features. All models were trained using default hyperparameters to maintain comparability and reproducibility. In both cohorts, receiver operating characteristic (ROC) curves and corresponding 95% confidence intervals were generated to evaluate model performance. The optimal model was selected based on the highest consistency between training and testing cohorts, as indicated by the smallest difference in Area Under the Receiver Operating Characteristic curve (AUROC). Using this model as a foundation, three additional models were constructed in the testing cohort: a clinical model, a radiomic model, and a fused model integrating both feature types. Model performance was comprehensively evaluated using AUROC as well as standard classification metrics, including accuracy, precision, recall, F1-score, and sensitivity. Calibration was assessed using calibration curves and Brier scores, while clinical utility was examined through decision curve analysis (DCA). To further interpret the combined model, Shapley Additive Explanations (SHAP) were applied, quantifying the importance of each feature and the direction of its contribution to the assessment of cognitive impairment.

### Preprocessing of data

Before analysis, missing values for each variable were examined. Since the proportion of missing data was low and randomly distributed across variables, cases with incomplete information were excluded from the study rather than imputed. This approach ensured that the final dataset included only samples with complete data for all variables.

### Statistical analysis

Baseline characteristics were compared between the training and testing cohorts. The Kolmogorov–Smirnov test was used to assess the normality of continuous variables. Normally distributed data were compared using the t-test and are presented as mean ± standard deviation, while non-normally distributed data were analyzed using the Mann–Whitney U test and are expressed as median (interquartile range). Categorical variables were analyzed using the chi-square test or Fisher’s exact test. A p-value < 0.05 (two-tailed) was considered to indicate statistical significance.

All statistical analyses were conducted using SPSS version 27.0, while model development and evaluation were performed using the scikit-learn library (version 1.2.2) in Python.

## Results

### Study participants

**Fig. 2:**
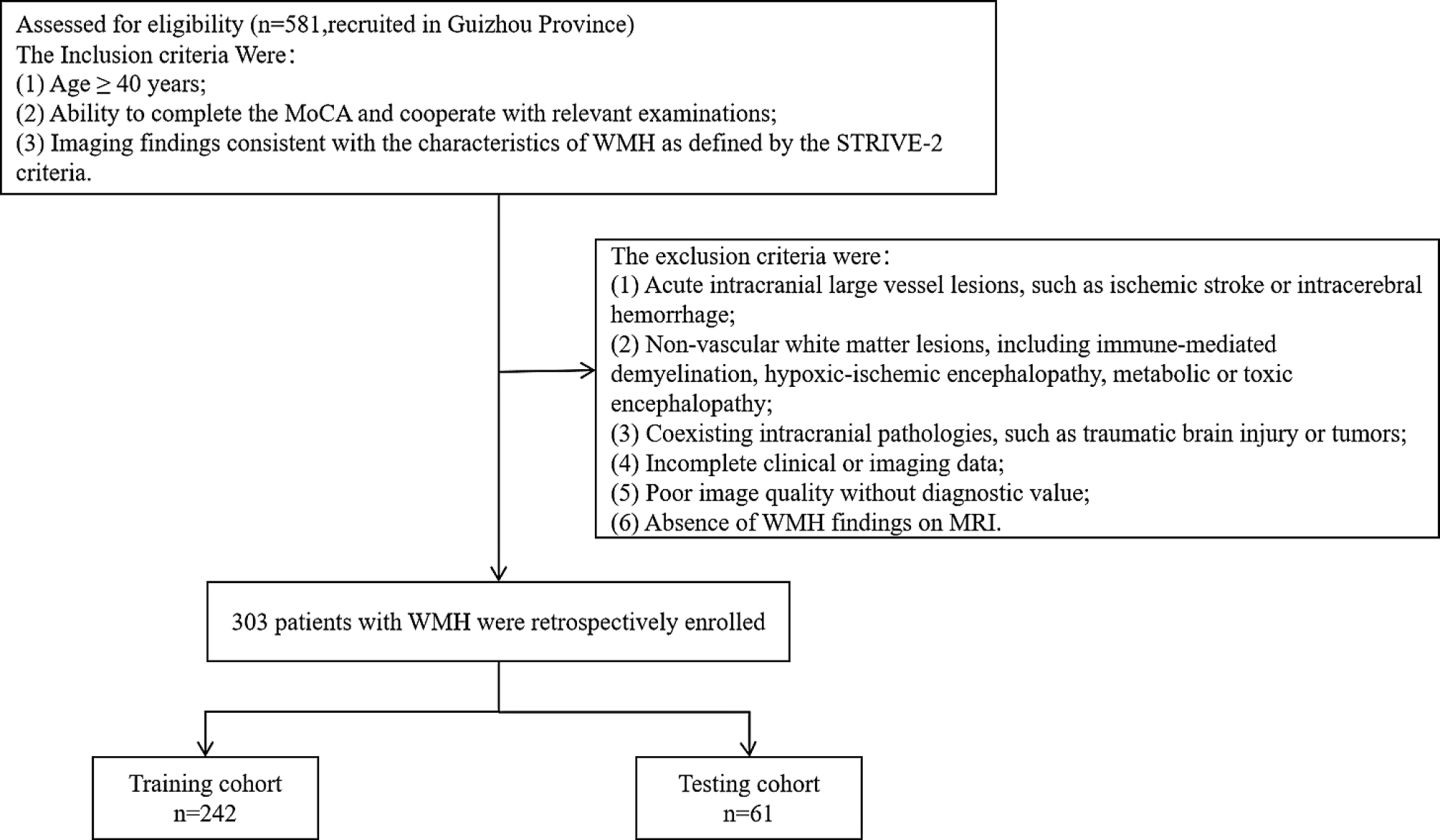
Flowchart of the inclusion and exclusion criteria for patient selection

After applying a series of inclusion and exclusion criteria, a total of 303 individuals with WMH were enrolled in this study (Fig. 2), including 120 males (39.6%) and 183 females (60.4%). Among them, 140 (46.2%) exhibited cognitive impairment, while 163 (53.8%) were cognitively intact. Of the total cohort, 242 participants formed the training cohort, and 61 constituted the testing cohort. Baseline characteristics of the training and testing cohorts are summarized in Table 1. Statistical analysis revealed no significant differences in baseline characteristics between the two groups (*p* > 0.05).

**Table 1:** Baseline characteristics of the training and testing cohorts

Group	Total (n = 303)	Training cohort (n = 242)	Testing cohort (n = 61)	P
Sex, n (%)				0.131
Male	120(39.6%)	101(41.7%)	19(31.1%)	
Female	183(60.4%)	141(58.3%)	42(68.9%)	
Weekly fruit intake, n (%)				0.696
≤2d/W	154(50.8%)	122(50.4%)	32(52.5%)	
3-4d/W	62(20.5%)	48(19.8%)	14(23.0%)	
≥5d/W	87(28.7%)	72(29.8)	15(24.6%)	
History of hypertension, n (%)				0.913
NO	167(55.1%)	133(55.0%)	34(55.7%)	
YES	136(44.9%)	109(45.0%)	27(44.3%)	
History of diabetes, n (%)				0.909
NO	272(89.8%)	217(89.7%)	55(90.2%)	
YES	31(10.2%)	25(10.3%)	6(9.8%)	
Smoking history, n (%)				0.304
NO	228(75.2%)	179(74.0%)	49(80.3%)	
YES	75(24.8%)	63(26.0%)	12(19.7%)	
Alcohol consumption history, n (%)				0.518
Moderate drinking	45(14.9%)	38(15.7%)	7(11.5%)	
No drinking	237(78.2%)	186(76.9%)	51(83.6%)	
Binge drinking	21(6.9%)	18(7.4%)	3(4.9%)	
Exercise habits, n (%)				0.111
Regularly	232(76.6%)	190(78.5%)	42(68.9%)	
Lack	71(23.4%)	52(21.5%)	19(31.1%)	
Weekly vegetable intake, n (%)				0.583
≤2d/W	15(5.0%)	12(5.0%)	3(4.9%)	
3-4d/W	75(24.8%)	63(26.0%)	12(19.7%)	
≥5d/W	213(70.3%)	167(69.0%)	46(75.4%)	
Napping habits, n (%)				0.055
YES	112(37.0%)	83(34.3%)	29(47.5%)	
NO	191(63.0%)	159(65.7%)	32(52.5%)	
Staying up late, n (%)				0.720
YES	31(10.2%)	24(9.9%)	7(11.5%)	
NO	272(89.8%)	218(90.1%)	54(88.5%)	
Age, years (median, IQR)	66.00(59.00, 72.00)	66.00(59.00, 72.00)	65.00(57.00, 73.00)	0.653
BMI, kg/m^2^ (median, IQR)	25.32(23.12, 27.92)	25.30(23.06, 27.95)	25.34(23.26, 28.19)	0.754
SBP, mmHg (median, IQR)	151.00(137.00, 170.00)	152.00(139.00, 171.00)	147.00(135.00, 165.00)	0.295
DBP, mmHg (median, IQR)	87.00(78.00, 94.00)	87.00(78.00, 94.00)	87.00(80.00, 95.00)	0.556
FBG, mmol/L (median, IQR)	4.84(4.41, 5.60)	4.85(4.42, 5.66)	4.70(4.31, 5.27)	0.327
Cr, μmol/L (median, IQR)	66.00(56.00, 79.00)	67.00(56.00, 81.00)	65.00(59.00, 75.00)	0.532
Hcy, μmol/L (median, IQR)	18.84(14.32, 26.35)	19.16(14.48, 27.06)	18.21(13.63, 24.88)	0.228
TyG index, (median, IQR)	8.80(8.41, 9.18)	8.81(8.47, 9.17)	8.72(8.30, 9.22)	0.494
Non-HDLC, mmol/L (media, IQR)	3.67(3.03, 4.31)	3.72(3.06, 4.31)	3.51(2.82, 4.32)	0.219
LDL-C, mmol/L (media, IQR)	3.22(2.60, 3.90)	2.26(2.66, 3.92)	3.11(2.43, 3.67)	0.099
HDL-C, mmol/L (media, IQR)	1.40(1.19, 1.66)	1.39(1.18, 1.64)	1.43(1.20, 1.72)	0.677
TC, mmol/L (median, IQR)	5.17(4.51, 5.74)	5.18(4.59, 5.75)	5.10(4.16, 5.72)	0.267
AC, cm (median, IQR)	91.00(85.00, 99.00)	91.00(84.00, 99.00)	92.00(87.00, 100.00)	0.533
UA, μmol/L (median, IQR)	328.00(270.00, 402.00)	325(271.00, 405.00)	333.00(261.00, 393.00)	0.750
TG, mmol/L (median, IQR)	1.62(1.16, 2.23)	1.66(1.18, 2.15)	1.57(1.06, 2.47)	0.582
Cognitive impairment, n (%)				0.958
No	163(53.8%)	130(53.7%)	33(54.1%)	
YES	140(46.2%)	112(46.3%)	28(45.9%)	

### Selection of features

For non-radiomic features, we employed RFE for feature selection. Based on the RFE results, ten non-radiomic features were retained: age, sex, BMI, SBP, FBG, Cr, Hcy, TyG index, non-HDL-C, and weekly fruit intake. For radiomic features, a total of 851 features were automatically extracted from WMH regions on FLAIR images using the Pyradiomics package. These included 14 shape features, 18 first-order features, 75 texture features, and 744 wavelet-transformed features. Feature selection was performed using the LASSO method, resulting in five relevant radiomic features: wavelet-LLH_ngtdm_Busyness, wavelet-LLL_gldm_LargeDependenceHighGrayLevelEmphasis, wavelet-LHL_ngtdm_Busyness, wavelet-HLL_glcm_DifferenceVariance, and wavelet-HLH_glcm_ClusterShade. These 15 selected features were subsequently input into six ML algorithms for further performance comparison.

### Multiple machine learning model performance

**Fig. 3:**
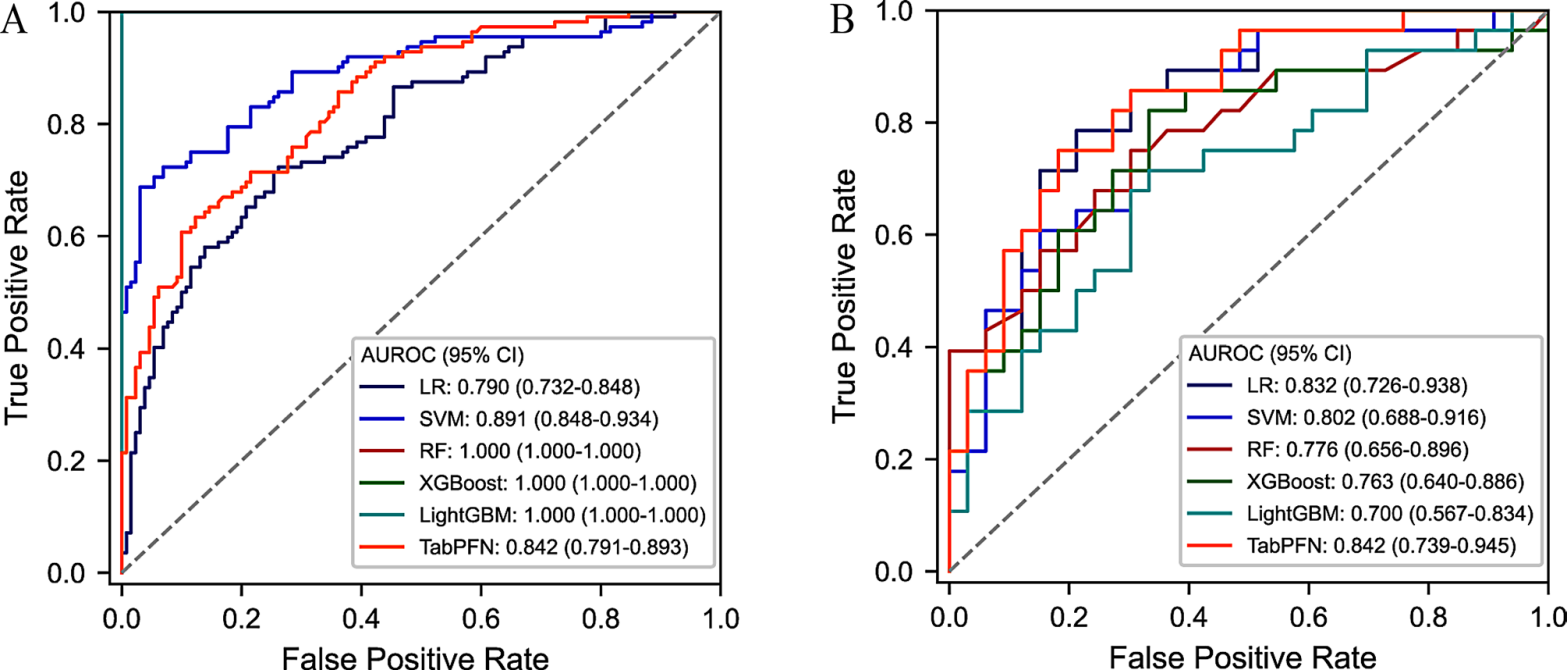
Receiver operating characteristic curves of six machine learning models in the training (**A**) and testing cohorts (**B**). (**A**) Receiver operating characteristic curves of the training cohort, where RF, XGBoost, and LightGBM models are overfitted, causing their Receiver operating characteristic curves to overlap as a single curve; therefore, only four Receiver operating characteristic curves are shown in the figure. Abbreviations: 95%CI, 95% confidence interval

Based on the 15 selected features, six machine learning models were constructed, including LR, SVM, RF, XGBoost, TabPFN, and LightGBM. The performance of the six ML models differed between the training and testing cohorts, as illustrated in Fig. 3, where Fig. 3A corresponds to the training cohort and Fig. 3B to the testing cohort. Overall, the TabPFN model demonstrated highly consistent ROC curve patterns across both datasets. The AUROC was 0.842 (95% confidence interval: 0.791–0.893) in the training cohort and 0.842 (95% confidence interval: 0.739–0.945) in the testing cohort, indicating excellent generalizability and minimal risk of overfitting. In addition, a key advantage of TabPFN is its ability to maintain stable performance without the need for hyperparameter tuning, which substantially reduces performance variability and enhances the reproducibility of results [31, 32]. In contrast, the three tree-based ensemble algorithms, including RF, LightGBM, and XGBoost, showed greater discrepancies between the training and testing cohorts, with their ROC curves indicating a clear tendency toward overfitting. Although ensemble models possess strong feature learning capabilities, their complex tree structures are prone to overfitting in high-dimensional data. Moreover, their performance often depends on extensive hyperparameter tuning to balance bias and variance, thereby increasing modeling complexity. In addition, the SVM model performed well in the training cohort (AUROC: 0.891) but showed a marked decline in the testing cohort (AUROC: 0.802), indicating a potential risk of overfitting due to the large performance gap between the two cohorts. Similarly, the logistic regression (LR) model exhibited a considerable discrepancy between the training (AUROC: 0.790) and testing (AUROC: 0.832) cohorts.

Therefore, based on the overall performance, TabPFN was selected as the optimal model for subsequent analyses.

### Evaluation of a single model

**Fig. 4:**
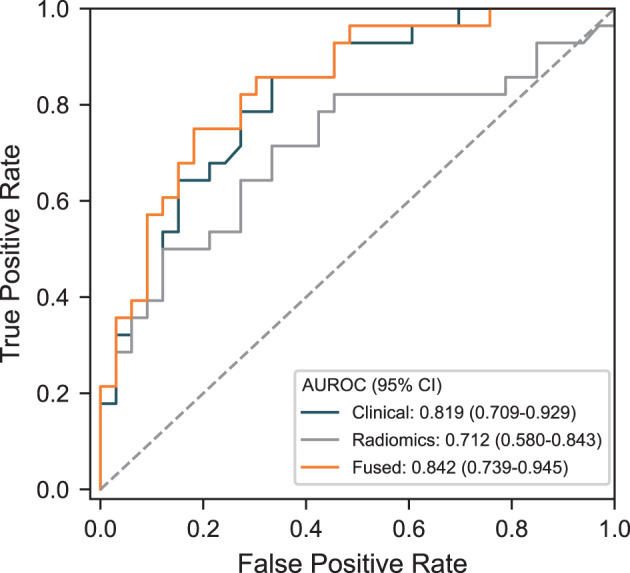
Receiver operating characteristic curves illustrating the classification performance of the clinical, radiomics, and fused models developed based on the TabPFN model on the testing cohort. Abbreviations: fused, clinical–radiomics combined model; 95%CI, 95% confidence interval

**Fig. 5:**
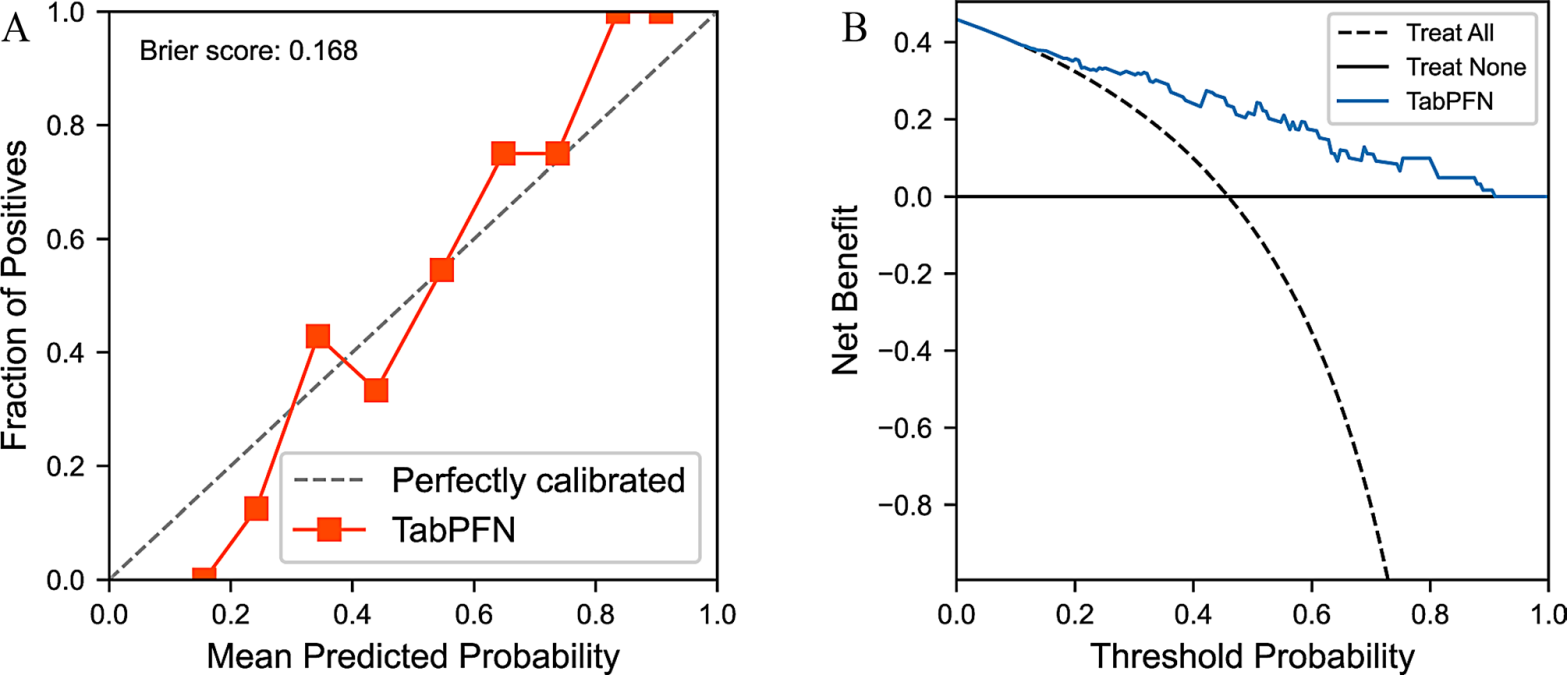
Calibration and decision curves of the clinical-radiomics combined model based on the TabPFN algorithm. (**A**) Calibration curve, where the x-axis represents the mean predicted probability and the y-axis represents the observed proportion of positive cases. The red solid line indicates the model’s performance, and the gray dashed line denotes perfect calibration. (**B**) Decision curve analysis (DCA), where the x-axis represents the threshold probability and the y-axis represents the net benefit. The black solid and dashed lines indicate the “treat none” and “treat all” strategies, respectively, and the blue solid line represents the TabPFN model

Based on the above results, we selected the TabPFN algorithm to construct the clinical model, the radiomics model, and the clinical-radiomics combined model for comparative analysis. As shown in Fig. 4, the ROC curves of the three models are presented, with the clinical-radiomics combined model (Fused) achieving an AUROC of 0.842 (95% confidence interval: 0.739–0.945), indicating superior classification performance compared to the clinical model (AUROC: 0.819, 95% confidence interval: 0.709–0.929) or radiomics model (AUROC: 0.712, 95% confidence interval: 0.580–0.843) alone. The performance metrics of the three models are summarized in Table 2. Among them, the clinical-radiomics fused model (Fused) achieved an F1-score of 0.737, an accuracy of 0.754, a recall of 0.750, a precision of 0.724, a sensitivity of 0.750, and a specificity of 0.758, all of which outperformed the single-modality models. In addition, we evaluated the calibration and decision curves of the combined TabPFN clinical and radiomic model. The TabPFN model exhibited good calibration and clinical utility. As shown in Fig. 5A, the predicted probabilities closely matched the observed outcomes (Brier score = 0.168), indicating reliable risk estimation. In addition, decision curve analysis (Fig. 5B) demonstrated that the model achieved higher net benefit than both “treat-all” and “treat-none” strategies across a broad range of threshold probabilities, suggesting its potential value for clinical decision-making. In summary, the TabPFN algorithm demonstrated favorable performance in constructing the clinical, radiomics, and combined models, with the combined model showing the best classification performance.

**Table 2:** Classification performance metrics of the clinical, radiomics, and fused models based on the TabPFN model on the testing cohort

	Clinical	Radiomics	Fused
F1 Score	0.691	0.583	0.737
Accuracy	0.721	0.672	0.754
Recall	0.679	0.500	0.750
Precision	0.704	0.700	0.724
AUROC	0.819	0.712	0.842
Sensitivity	0.679	0.500	0.750
Specificity	0.758	0.818	0.758

### Feature importance and feature interpretation

**Fig. 6:**
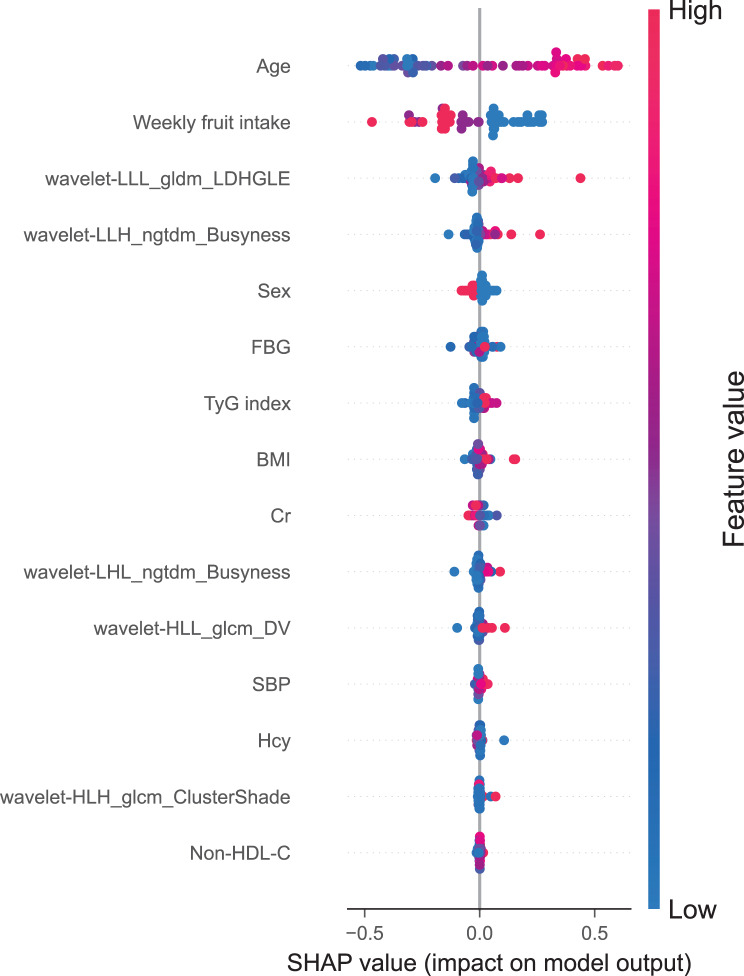
Shapley Additive exPlanations (SHAP) summary plot based on the optimal TabPFN model, showing the importance of each feature for cognitive impairment and its positive or negative effect. Abbreviations: wavelet-LLL_gldm_LDHGLE, wavelet-LLL_gldm_LargeDependenceHighGrayLevelEmphasis; wavelet-HLL_glcm_DV, wavelet-HLL_glcm_DifferenceVariance

**Fig. 7:**
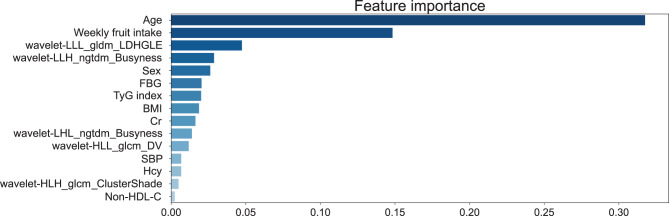
Feature importance bar chart based on the optimal TabPFN model, showing the importance of each feature for cognitive impairment. Abbreviations: wavelet-LLL_gldm_LDHGLE, wavelet-LLL_gldm_LargeDependenceHighGrayLevelEmphasis; wavelet-HLL_glcm_DV, wavelet-HLL_glcm_DifferenceVariance

Based on the TabPFN model, we generated SHAP plots (Fig. 6) and feature importance bar charts (Fig. 7) to interpret the contribution of each feature to the model. These visualizations illustrate the impact of each feature on the predicted outcomes. Positive SHAP values (shown in red) indicate that higher feature values increase the probability of CI. In contrast, negative SHAP values (shown in blue) indicate that lower feature values decrease this probability. The analysis revealed that age, weekly fruit intake, wavelet-LLL_gldm_LargeDependenceHighGrayLevelEmphasis(wLLL_LDHGLE), wavelet-LLH_ngtdm_Busyness, and sex are the most important features for assessing CI. Higher values of these features are associated with a higher risk.

## Discussion

In this study, we developed a TabPFN-based ML model that integrates radiomic features of WMH with clinical variables to assess CI. The multimodal approach achieved superior performance compared with single-modality models, demonstrating strong discriminative ability, good calibration, and promising clinical applicability. These results suggest that integrating WMH-derived radiomic signatures with routine clinical variables may enhance the clinical utility of early identification of CI in at-risk populations. Among these, TabPFN, as an emerging ML method, offers advantages such as high computational efficiency and strong generalization capability [32]. The TabPFN algorithm not only achieved excellent performance in the training cohort but also showed minimal discrepancy in the testing cohort, with an AUROC of 0.842, indicating strong generalization and robust evaluation performance. Additionally, decision curve and calibration analyses further confirmed the clinical utility of the combined model.

Several prior studies have employed ML approaches to evaluate CI, providing context for the design and advantages of our multimodal model. For instance, a recent investigation in patients with CSVD found that higher volume of WMH was independently associated with cognitive dysfunction in an LR framework [33]. However, such volume-based models typically ignore the microstructural heterogeneity of WMH (e.g., shape, texture, spatial dependencies) that may better reflect underlying pathology. Another study applying MRI radiomics to WMH regions reported that combined radiomic and clinical models achieved excellent discrimination (AUC = 0.900) in detecting CI in WMH patients, but it was limited by a small sample size and absence of external validation [21]. In contrast, our study analyzed a larger cohort, enhancing model stability and generalizability, although further external validation is warranted. In addition, some studies that focus solely on WMH volume changes or demographic and vascular risk factors often overlook the microstructural heterogeneity of WMH (e.g., texture) and modifiable lifestyle factors [34, 35]. Petersen et al. demonstrated that considering WMH-related network connectivity significantly improves the prediction of cognitive performance compared with conventional WMH volume alone. This finding highlights that the microstructural and spatial characteristics of WMH, rather than volumetric measures alone, contribute importantly to cognitive outcomes, supporting the rationale for incorporating radiomic features in assessment models [36]. In line with this, our study integrates WMH radiomic signatures with clinical variables, capturing both structural heterogeneity and systemic risk factors, thereby enhancing model interpretability and assessment performance relative to previous volume-based or single-modality approaches. Based on these findings, we examined the main features influencing the model’s evaluation of CI, aiming to enhance interpretability and highlight important risk factors in our study population. Feature selection using RFE and LASSO identified age, weekly fruit intake, the radiomic features wLLL_LDHGLE and waveletLLH_ngtdm_Busyness, and sex as the most important features of CI in our cohort. To enhance interpretability, we further applied SHAP to quantify and visualize the contribution of each feature to the output of the CI assessment model.

Through model interpretability analysis, Age and weekly fruit intake showed a strong correlation with CI: increased age was associated with a higher risk of CI, while higher fruit intake effectively reduced the risk. These findings align with previous studies [37–42], potentially due to age-related vascular degeneration that increases WMH burden, impairing cerebral blood flow and nutrient supply, thereby affecting cognitive function [43]. Notably, increased fruit intake was associated with a reduced risk of CI, indicating a potential protective role. Although previous studies have primarily focused on vascular and demographic factors associated with cognitive decline, the impact of dietary habits, particularly fruit intake, has been relatively understudied. This makes our findings both novel and clinically relevant. Fruits are rich in bioactive compounds such as flavonoids, polyphenols, and antioxidants, which have been shown to improve endothelial function, reduce oxidative stress, and modulate neuroinflammation, thereby preserving both cerebral microstructure and vascular integrity. Epidemiological studies and randomized trials have demonstrated that higher fruit or berry consumption is associated with reduced WMH burden, improved cognitive performance, and lower risk of dementia and CI [42, 44–46]. This finding suggests that dietary modification may serve as a practical and effective approach to slowing cognitive decline, reinforcing its role in preventive strategies. Among the radiomic features, wLLL_LDHGLE and wavelet-LLH_ngtdm_Busyness were significantly associated with the model’s evaluation of CI. A higher wLLL_LDHGLE value indicates the presence of large, confluent areas of high signal intensity within the WMH, rather than scattered punctate foci [47, 48]. Such patterns likely reflect more advanced white-matter damage, including demyelination, gliosis, or interstitial fluid accumulation, which could disrupt long-range axonal connectivity and contribute to cognitive decline [49, 50]. In contrast, the wavelet-LLH_ngtdm_Busyness feature quantifies the rate of gray-level change between neighboring voxels. Higher values suggest greater texture heterogeneity and irregular lesion boundaries, capturing internal complexity within the WMH [47, 48]. Overall, these distinct radiomic features provide a refined characterization of lesion extent and internal heterogeneity, enabling a more accurate assessment of white-matter integrity and its relationship with CI. Previous studies have also indicated that radiomic features describing lesion continuity and internal heterogeneity provide valuable information beyond conventional WMH volume in studies assessing cognitive outcomes [22, 51, 52]. These features capture the microstructural complexity and tissue heterogeneity of lesions, thereby contributing to a more precise prediction of cognitive decline. These results underscore that radiomic features can extend beyond traditional WMH volume measurements, providing more comprehensive and detailed pathological information for assessing cognitive decline.

Additionally, our findings revealed that females have a higher risk of CI than males, consistent with previous findings [53, 54]. This may relate to the critical role of estrogen in maintaining cerebral vascular health; postmenopausal estrogen decline accelerates cerebrovascular aging, leading to WMH accumulation and cognitive decline [55, 56]. Our study also demonstrated that a high TyG index level is associated with an increased risk of cognitive impairment, which may be related to the insulin resistance status reflected by the TyG index. The TyG index is a reliable marker for insulin resistance, which is closely associated with metabolic syndrome, atherosclerosis, cerebrovascular dysfunction, and neurodegeneration. High TyG levels can damage cerebral microvasculature, promoting WMH formation, thereby impairing cerebral nutrient supply and metabolism, leading to cognitive impairment [57–59]. Therefore, the TyG index, as an easily obtainable and cost-effective biomarker, also holds significant value in assessing the risk of CI.

In this study, elevated Cr levels were negatively associated with the risk of CI. This contrasts with the majority of previous studies, which have reported that elevated Cr is associated with an increased risk of CI, particularly among patients with chronic kidney disease, those on dialysis, or individuals with comorbid cardiovascular or metabolic conditions [60–62]. However, some studies in community-dwelling older adults or individuals at early risk of CI have also found that higher Cr may be linked to relatively better cognitive function [63]. In our study cohort, which included community-dwelling older adults and early CI high-risk individuals with generally preserved renal function, variations in Cr may reflect factors such as muscle mass and nutritional status, which themselves are associated with better cognitive outcomes. Therefore, in ordinary older adults, Cr may serve not only as a marker of kidney function but also as an indicator of overall health status. These observations suggest that the relationship between Cr and CI may depend on the characteristics of the study population and may not follow a simple linear pattern. Future studies incorporating assessments of muscle mass, nutritional status, and longitudinal follow-up are warranted to further explore this potential mechanism.

## Limitations

Despite the promising performance of the proposed model, several limitations should be acknowledged. First, this was a single-center study conducted in a socioeconomically underdeveloped region of Guizhou Province, where participants generally had relatively low educational levels, which may limit the generalizability of the findings. Second, the total sample size is relatively limited, specifically the independent test set, which may constrain the statistical power of our evaluation. Moreover, external validation of the findings was not performed. Future multicenter, large-scale, and longitudinal studies are warranted to expand the sample size, verify the robustness, and validate the clinical utility of the model in tracking cognitive trajectories and guiding early interventions. In addition, only FLAIR-derived radiomic features were analyzed, and the absence of multimodal MRI data (e.g., DTI or fMRI) restricted the exploration of microstructural and functional mechanisms. Finally, although the SHAP analysis provided insights into feature importance, further biological and clinical validation is required to elucidate the potential mechanisms through which radiomic and clinical features jointly contribute to CI.

## Conclusion

This study demonstrates that integrating radiomic features of WMH with clinical features using a TabPFN-based ML framework can substantially improve the accuracy and clinical utility of CI assessment. Taken together, our findings highlight an interpretable and clinically applicable paradigm that may facilitate earlier identification and more personalized management of CI.

## Data Availability

The data that support the findings of this study are available from the corresponding author, upon reasonable request.

## Abbreviations

WMH: White matter hyperintensities
MoCA: Montreal Cognitive Assessment
MMSE: Mini-Mental State Examination
SHAP: SHapley Additive exPlanations
CI: Cognitive impairment
AD: Alzheimer’s Disease
MRI: Magnetic Resonance Imaging
CT: Computed tomography
CSVD: Cerebral small vessel disease
ML: Machine learning
SVM: Support vector machines
RF: Random forests
LR: Logistic regression
AC: Abdominal circumference
SBP: Systolic blood pressure
DBP: Diastolic blood pressure
FBG: Fasting blood glucose
TG: Triglyceride
TC: Total cholesterol
LDL-C: Low-density lipoprotein cholesterol
HDL-C: High-density lipoprotein cholesterol
Hcy: Homocysteine
UA: Uric acid
Cr: Creatinine
TyG index: Triglyceride glucose index
BMI: Body mass index
Non-HDL-C: Non-high-density lipoprotein Cholesterol
FLAIR: Fluid-Attenuated Inversion Recovery
RFB: Receptive Field Block
LASSO: Least absolute shrinkage and selection operator
RFE: Recursive feature elimination
XGBoost: Extreme Gradient Boosting
TabPFN: Tabular Prior-Data Fitted Networks
LightGBM: Light Gradient Boosting Machine
ROC: Receiver operating characteristic
AUROC: Area under the receiver operating characteristic curve
DCA: Decision curve analysis
